# Precipitation Hardening in Ferroelectric Ceramics

**DOI:** 10.1002/adma.202102421

**Published:** 2021-07-24

**Authors:** Changhao Zhao, Shuang Gao, Tiannan Yang, Michael Scherer, Jan Schultheiß, Dennis Meier, Xiaoli Tan, Hans‐Joachim Kleebe, Long‐Qing Chen, Jurij Koruza, Jürgen Rödel

**Affiliations:** ^1^ Department of Materials and Earth Sciences Nonmetallic Inorganic Materials Technical University of Darmstadt Alarich‐Weiss‐Straße 2 64287 Darmstadt Germany; ^2^ Materials Research Institute and Department of Materials Science and Engineering The Pennsylvania State University University Park PA 16802 USA; ^3^ Department of Materials Science and Engineering Norwegian University of Science and Technology Trondheim 7034 Norway; ^4^ Department of Materials Science and Engineering Iowa State University Ames IA 50011 USA

**Keywords:** dielectrics, electromechanical hardening, ferroelectrics, mechanical quality factor, precipitation

## Abstract

Domain wall motion in ferroics, similar to dislocation motion in metals, can be tuned by well‐concepted microstructural elements. In demanding high‐power applications of piezoelectric materials, the domain wall motion is considered as a lossy hysteretic mechanism that should be restricted. Current applications for so‐called hard piezoelectrics are abundant and hinge on the use of an acceptor‐doping scheme. However, this mechanism features severe limitations due to enhanced mobility of oxygen vacancies at moderate temperatures. By analogy with metal technology, the authors present here a new solution for electroceramics, where precipitates are utilized to pin domain walls and improve piezoelectric properties. Through a sequence of sintering, nucleation, and precipitate growth, intragranular precipitates leading to a fine domain structure are developed as shown by transmission electron microscopy, piezoresponse force microscopy, and phase‐field simulation. This structure impedes the domain wall motion as elucidated by electromechanical characterization. As a result, the mechanical quality factor is increased by ≈50% and the hysteresis in electrostrain is suppressed considerably. This is even achieved with slightly increased piezoelectric coefficient and electromechanical coupling factor. This novel process can be smoothly implemented in industrial production processes and is accessible to simple laboratory experimentation for microstructure optimization and implementation in various ferroelectric systems.

## Graphical abstract

A novel ferroelectric hardening method is proposed by precipitating a secondary phase inside of the ferroelectric grains. Akin to hindered dislocation motion in metals, domain wall motion in ferroelectrics is impeded and loss is reduced. The mechanical quality factor in the model system BaTiO_3_–CaTiO_3_ is enhanced by 1.5 times, while the piezoelectric coefficient and planar electromechanical coupling factor remain nearly unchanged.

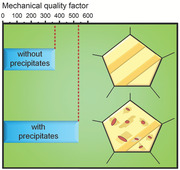

## 1 Introduction

Piezoelectricity is an important feature of poled ferroelectrics, which enables conversion between electrical and mechanical signals. Ferroelectric materials are therefore widely utilized in actuators, transducers, sensors, *etc*.^[^ ^1^ ^]^ With the development of technology, ferroelectric materials are becoming increasingly important in novel and highly demanding application fields, for example, photon–electronic communication^[^^2,3]^ and energy storage.^[^ ^4^ ^]^

The macroscopic electromechanical response of piezoelectrics relies on an interplay of intrinsic and extrinsic contributions, where the intrinsic effect utilizes reversible lattice extension/contraction and the extrinsic effect is facilitated by irreversible, hysteretic domain wall motion and possibly phase transition.^[^ ^5^ ^]^ High‐power applications in ultrasonic motors, transducers, and transformers demand so‐called hard ferroelectrics with low energy losses.^[^ ^6^ ^]^ Therefore, a stringent reduction of all lossy electrical and mechanical mechanisms, in particular an effective ferroelectric domain wall immobilization, is required. The state‐of‐the‐art concept for hardening of ferroelectrics relies on doping with acceptor elements.^[^ ^7^, ^8^ ^]^ The effectiveness of this approach is quantified by the mechanical quality factor *Q*_m_, which is the reciprocal of mechanical loss and places a stark requirement on resonance applications.^[^ ^1^, ^9^, ^10^ ^]^ The mechanism of acceptor doping relies on oxygen vacancies, which become mobile at moderate temperature with the consequence that the market‐dominating material, lead zirconate titanate (PZT), heats up under high vibration velocity^[^ ^8^ ^]^ and loses 50% of its electromechanical quality factor already at a moderate usage temperature of 79 °C.^[^ ^11^ ^]^ This effect decisively limits the operational range of piezoceramics.

Ferroelectric hardening as a process to reduce hysteretic movement of domain walls (2D carriers of deformation) is suggested to bear strong resemblance to hardening of metals, where the mobility of dislocations (1D carriers of deformation) is reduced by multidimensional defects. Hardening or strengthening of metals is achieved by point defects (0D defects), dislocations (1D defects), grain boundaries (2D defects), and secondary phases (3D defects in form of precipitates or added secondary phases).^[^ ^12^ ^]^ Precipitation hardening in metals is particularly appealing as it affords high homogeneity and efficient industry‐scale processing, both for applications as structural materials^[^ ^13^, ^14^, ^15^ ^]^ and as ferromagnetic materials.^[^ ^16^, ^17^, ^18^ ^]^

Recently, secondary phase ferroelectric hardening was demonstrated in Na_1/2_Bi_1/2_TiO_3_‐based (NBT‐based) piezoceramics by forming 0–3 type composites of 0.94Na_1/2_Bi_1/2_TiO_3_‐0.06BaTiO_3_ (NBT‐6BT) matrix and added ZnO grains, located at the grain boundaries.^[^ ^19^, ^20^, ^21^, ^22^, ^23^ ^]^ A lower dielectric loss and a nearly twofold increase in *Q*_m_ were found in a NBT‐6BT:0.1ZnO composite.^[^ ^19^ ^]^ The hardening mechanism has recently been rationalized through a mechanical interaction between the secondary‐phase particles and the matrix.^[^ ^19^, ^21^ ^]^ Riemer et al. pointed out that the difference in the thermal expansion coefficient of ZnO and NBT‐BT grains induces deviatoric stresses in the matrix, which stabilize the ferroelectric phase.^[^ ^21^ ^]^ The hardening effect through this composite approach was also observed in other piezoelectric systems such as 0.83(Na_1/2_Bi_1/2_)TiO_3_‐0.17(K_1/2_Bi_1/2_)TiO_3_,^[^ ^22^ ^]^ Bi_3_TaTiO_9_:40 wt%BiFeO_3_,^[^ ^24^ ^]^ and 0.2Pb(Zn_1/3_Nb_2/3_)O_3_‐0.8Pb(Zr_0.5_Ti_0.5_)O_3_.^[^ ^25^ ^]^

An apparent shortcoming of the NBT‐BT:ZnO composites lies in the limited flexibility of tuning microstructure. Due to the nature of the composite processing, the ZnO grains are predominantly located at grain boundaries and triple junctions, which limit their interaction with ferroelectric domains located inside the matrix grains. Inspired by precipitation hardening in metals, we hypothesize that precipitation can be used as a means to homogeneously distribute secondary‐phase particles into ferroelectric grains, in order to alter the domain structure, pin domain walls, and suppress their motion. Despite the extensive investigations on precipitation hardening on metals, the studies on ceramic materials are quite limited. One successful case of precipitate‐tuned ceramic material is Mg partially stabilized ZrO_2_ (Mg‐PSZ), in which the fracture toughness has a fourfold increase and achieves 10 MPa m^1/2^.^[^ ^26^ ^]^ Besides, this concept has also been previously applied in mere exploratory fashion to Al_2_O_3_‐Fe_2_O_3_^[^ ^27^ ^]^ and MgO‐Cr_2_O_3_^[^ ^28^ ^]^ solid solutions, both focused on tuning the mechanical properties. To the best of our knowledge, precipitation has not been utilized in electroceramics so far.

Here, we demonstrate that secondary‐phase precipitates can be applied to tune the domain size and to pin domain walls, which effectively hardens the electromechanical response—a mechanism hereafter referred to as “precipitation hardening.” The concept is demonstrated using the model pseudo‐binary system BaTiO_3_‐CaTiO_3_, (barium calcium titanate, BCT), with a curved line of solid solubility—a precondition for precipitation in solid solutions. Non‐ferroelectric CaTiO_3_‐rich precipitates were successfully introduced in the ferroelectric BaTiO_3_‐rich matrix. Through various structural and microstructural characterizations and electrical property measurements, it was found that the precipitates have an influence on their vicinal domain structures and suppress domain switching during the application of electric field, leading to lower saturated polarization, strain, permittivity at a poled state, and a higher mechanical quality factor.

## 2 Experimental Section

### 2.1 Sample Preparation

**Figure 1:**
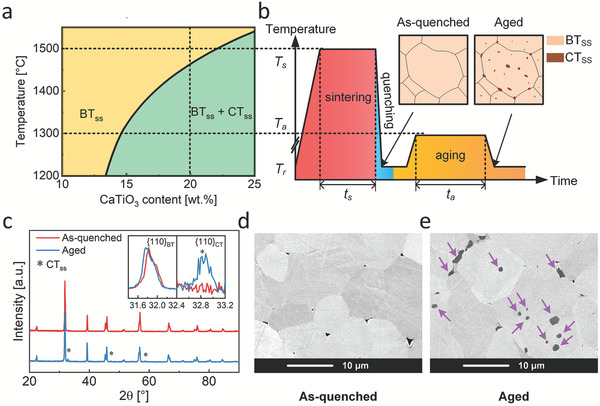
a) Phase diagram of the BCT solid solution.^[^ ^37^ ^]^ BT_ss_ and CT_ss_ represent the Ba‐rich and Ca‐rich solid solution phases, respectively. b) Temperature profile of the precipitate‐formation process: sintering, quenching, and aging, where *T*_s_, *T*_a_, and *T*_r_ represent the sintering temperature, aging temperature, and room temperature, respectively, and *t*_s_ and *t*_a_ represent the sintering duration and aging duration, respectively. Schematics of the microstructures after quenching and aging are depicted in the insets. c) XRD patterns of the as‐quenched and of the sample aged at 1325 °C for 8 h. The reflections arising from the CT_ss_ precipitates are marked by asterisks. d,e) SEM images of unaged (S_u_) and two‐stage aged (S_t_) samples. Dark features in (d) were identified as triple‐point pores, while dark grey areas in (e) were identified to be the CT_ss_ precipitates (see the purple arrows).

The BCT20 samples were synthesized by the solid‐state reaction method. Starting powders of BaCO_3_ (Alfa Aesar, 99.95%), CaCO_3_ (Alfa Aesar, 99.99%), and TiO_2_ (Anatase, Alfa Aesar, 99.6%) were weighed according to stoichiometry. The powder mixture was ball milled at 250 r min^−1^ for 12 h in ethanol. After drying, calcination was performed at 1100 °C for 4 h. To ensure complete chemical reaction, powders were then crushed and ball milled again at 250 r min^−1^ for 12 h and calcined for a second time with the same condition as the first calcination. Then, the twice‐calcined powders were cold‐isostatically pressed into pellets with a dimension of ≈Ø 10 mm × 1 mm under a hydrostatic pressure of 357 MPa. The pellets were then sintered at 1500 °C, which is in the single‐phase region of the BCT phase diagram (**Figure** **1**a), for 8 h using a tube furnace. When sintering was completed, the samples were air‐quenched, that is, they were directly taken out of the tube furnace from 1500 °C to room temperature, to kinetically suppress the formation of the Ca‐rich secondary phase. Those as‐quenched samples (unaged samples) were denoted as S_u_. For the aging treatment, the as‐quenched samples were annealed at 1200 °C for 72 h and then cooled with 5 K min^−1^ and were denoted as S_o_. Some of the 1200 °C‐annealed samples were further annealed at 1300 °C for 24 h and were denoted as S_t_.

### 2.2 Microstructure Characterization

X‐ray diffraction (XRD) measurements were conducted using a laboratory XRD (Bruker D8 Advanced, Germany) with Cu Kα radiation. Bulk samples were ground with a 1200‐mesh sand paper followed by annealing at 300 °C for 2 h before investigation. Bragg–Brentano geometry was adopted. The two‐theta range from 10° to 90° with a step size of 0.02° was considered. The scanning electron microscopy (SEM) images were taken by (XL30FEG, Philips, Amsterdam, Netherlands). Samples were ground and polished with diamond polishing paste down to 0.25 μm particle size before the measurement. The back‐scattered electron (BSE) detector was used to distinguish between Ba‐rich and Ca‐rich phases. A relatively low electron energy of 8 keV was selected to visualize grain boundaries by the contrast difference of grains with different orientations.

The transmission electron microscopy (TEM) images and energy dispersive spectroscopy (EDS) mappings were taken by a JEM‐2100F TEM. To prepare the TEM sample, ceramic pellet was first ground to a thickness of 250 μm. Then, a disk with 3 mm in diameter was prepared by ultrasonic cutting for further polishing. Both the top and bottom surfaces of the disk were polished using diamond lapping films with grain size of 9, 6, 3, 1, and 0.25 μm in turn, to gradually reduce the thickness of the disk down to 20 μm. The polished 3 mm‐disk was then annealed at 400 °C for 0.5 h with both slow heating and cooling rate of 1 °C min^−1^ to release the accumulated strain during polishing. Afterwards, the disk was glued to a supporting molybdenum grid and finally thinned to obtain electron transparent areas by ion milling (Gatan Model 600 dual ion mill).

Piezoresponse force microscopy (PFM) data was recorded using a NT‐MDT (NTEGRA, Apeldoorn, The Netherlands) atomic force microscope. A conductive Ti/Ir coated tip (Asyelec.01‐R2, Oxford Instruments, USA) was used for scanning in contact mode. For domain imaging, a sinusoidal alternating current excitation voltage of 10 V was applied to the back electrode at a frequency of 40.13 kHz. The deflection of the laser signal was read out as the amplitude, *R*, and the phase, *ϑ*, of the piezoresponse using a lock‐in amplifier (SR830, Stanford Research Systems, USA). Spatial resolution of *R*cos*ϑ* enabled to qualitatively distinguish domains with different orientation.

### 2.3 Phase‐Field Simulation

Phase‐field simulations were performed on a 2D system consisting of a circular CaTiO_3_ particle with a diameter of 130 nm inside a BaTiO_3_ matrix. The ferroelectric domain structure was simulated by solving the time‐dependent Ginzburg–Landau equation^[^ ^29^ ^]^ for the evolution of the polarization field **P**(*x*), that is,

(1) $$\begin{array}{c} \frac{\partial P}{\partial t}\;\;=\;\;-L_{\text{P}}\frac{\partial F}{\partial P} \end{array}$$

A periodic boundary condition was employed. The free energy, *F*, was formulated as the sum of the Landau free energy, the electrostatic free energy, the elastic energy, and the gradient energy, that is, *F* = *F*_Landau_ + *F*_electrostatic_ + *F*_elastic_ + *F*_gradient_. The Landau free energy was given by

(2) $$\begin{array}{c} F_{\text{Landau}}=\int \left(a_{i}P_{i}^{2}+a_{ij}P_{i}^{2}P_{j}^{2}+a_{ijk}P_{i}^{2}P_{j}^{2}P_{k}^{2}+a_{ijkl}P_{i}^{2}P_{j}^{2}P_{k}^{2}P_{l}^{2}\right)\;\text{d}x^{3} \end{array}$$

where *a*_*i*_, *a*_*ij*_, *a*_ijk_, and *a*_*ijkl*_ were the Landau coefficients. An Einstein summation convention of automatic summation over repeated indices *i*, *j*, *k*, *l* = 1, 2, 3 was employed herein. The electrostatic energy was written as

(3) $$\begin{array}{c} F_{\text{electrostatic}}=\int \left(-\frac{1}{2}\kappa_{0}\kappa_{ij}^{b}E_{i}E_{j}-E_{i}P_{i}\right)\;\text{d}x^{3} \end{array}$$

where κ_0_ was the vacuum permittivity, **κ** ^*b*^ was the background dielectric constant, and **E**(*x*) was the electric field, which was obtained by solving the electrostatic equilibrium equation

(4) $$\begin{array}{c} \frac{\partial }{\partial x_{i}}\left(\kappa_{0}\kappa_{ij}^{b}E_{j}+P_{i}\right)=0 \end{array}$$

with a periodic boundary condition. The elastic energy was given by

(5) $$\begin{array}{c} F_{\text{elastic}}\;\;=\;\;\int \frac{1}{2}C_{ijkl}\left(\varepsilon_{ij}-\varepsilon_{ij}^{0}\right)\;\left(\varepsilon_{kl}-\varepsilon_{kl}^{0}\right)\;\text{d}x^{3} \end{array}$$

Here, C was the elastic stiffness tensor, **ε**(**x**) was the strain field, and **ε** ^0^(**x**) was the eigenstrain field given by $\varepsilon_{\text{ij}}^{0}\;\;=\;\;Q_{\text{ijkl}}\;P_{\text{k}}\;P_{\text{l}}$, with **Q** being the electrostrictive coefficient. The strain field was obtained by solving the elastic equilibrium equation

(6) $$\begin{array}{c} \frac{\partial \sigma_{ij}}{\partial x_{j}}=0,\;\sigma_{ij}=C_{ijkl}\left(\varepsilon_{kl}-\varepsilon_{kl}^{0}\right) \end{array}$$

where **σ** (**x**) was the stress field. A periodic boundary condition with a zero homogeneous stress was employed. The gradient energy was expressed as

(7) $$\begin{array}{c} F_{\text{gradient}}\;\;=\;\;\int \frac{1}{2}g_{ijkl}\frac{\partial P_{i}}{\partial x_{j}}\;\frac{\partial P_{k}}{\partial x_{l}}\;\text{d}x^{3} \end{array}$$

where *g* was the gradient energy coefficient.

The material constants of BaTiO_3_ used in the present work, including the Landau coefficients, the background dielectric constant, the elastic stiffness, the electrostrictive coefficient, and the gradient energy coefficient are listed in **Table** **1**. The CaTiO_3_ precipitate was considered as a dielectric particle with a dielectric constant of κ^*r*^ = 168^[^ ^30^ ^]^ and a corresponding Landau coefficient given by *a*_1_ = (2κ_0_κ^*r*^)^−1^ = 3.36 × 10^8^ J m C^−2^; higher‐order Landau coefficients were neglected. The background dielectric constant, the elastic stiffness, the electrostrictive coefficient, and the gradient energy coefficient of CaTiO_3_ were taken the same as those of BaTiO_3_ for simplicity. The system was discretized into square meshes with a mesh size of 0.8 nm × 0.8 nm for the numerical simulation.

**Table 1:** Material constants of BaTiO_3_

Constant	Value	Constant	Value
*a*_1_	− 3.712 × 10^7^ J m C^−2[^ ^31^ ^]^	$\kappa_{11}^{\text{b}}$	44^[^ ^32^ ^]^
*a*_11_	− 2.097 × 10^8^ J m^5^C^−4^	*c*_11_	1.78 × 10^11^ J m^–3[^ ^33^, ^34^ ^]^
*a*_12_	7.974 × 10^8^ J m^5^C^−4^	*c*_12_	0.96 × 10^11^ J m^–3^
*a*_111_	1.294 × 10^9^ J m^9^C^−6^	*c*_44_	1.22 × 10^11^ J m^–3^
*a*_112_	− 1.950 × 10^9^ J m^9^C^−6^	*Q*_11_	0.110 m^4^C^–2[^ ^35^ ^]^
*a*_123_	− 2.500 × 10^9^ J m^9^C^−6^	*Q*_12_	−0.045 m^4^C^–2^
*a*_1111_	3.863 × 10^10^ J m^13^C^−8^	*Q*_44_	−0.029 m^4^C^–2^
*a*_1112_	2.529 × 10^10^ J m^13^C^−8^	*g*_11_	5.0 × 10^–10^ J m^3^C^–2^
*a*_1122_	1.637 × 10^10^ J m^13^C^−8^	*g*_12_	0
*a*_1123_	1.367 × 10^10^ J m^13^C^−8^	*g*_44_	0.2 × 10^–10^ J m^3^C^–2^

### 2.4 Electrical Characterization

The samples were made in disk shape with a dimension of ≈Ø 8.00 mm × 0.50 mm. Both top and bottom surfaces were fully covered with Pt electrodes. For the measurements of unpoled samples, the samples were annealed at 300 °C for 2 h to release the polarized state and then were kept at room temperature for 24 h before the measurements. Poling was conducted at 4 kV mm^−1^ for 15 min at room temperature, followed by a 24 h period before investigation. Polarization and strain hysteresis loops were obtained by a modified Sawyer–Tower circuit and an optical displacement sensor (D63, Philtec Inc., USA). A triangular electric field with a maximum 2 kV mm^−1^ and frequency of 1 Hz for bipolar measurements and 2 Hz for unipolar measurements (to ensure the same ramping rate of the applied field) was applied. The permittivity frequency spectra were recorder by a broadband dielectric analyzer (Novocontrol, Germany). A sinusoidal alternating current signal with peak‐to‐peak voltage *V*_pp_ of 2.88 V was applied with frequency ranging from 10^−1^ to 10^5^ Hz. The longitudinal piezoelectric coefficient *d*_33_ was determined using a commercial Berlincourt meter (Piezotest PM300, Singapore), with a static clamping force of 2 N, dynamic driving force of 0.25 N, and driving frequency of 110 Hz. Planar coupling factor, *k*_p_, and mechanical quality factor, *Q*_m_, were quantified using the impedance spectrum as function of frequency near the resonance frequency. The impedance spectra were also obtained by the broadband dielectric analyzer (Novocontrol, Germany) and the driving voltage was set as *V*_pp_ = 0.288 V. *Q*_m_ was obtained by the following equation:^[^ ^36^ ^]^

(8) $$\begin{array}{c} Q_{\text{m}}=\frac{1}{2\pi f_{\text{r}}f_{\text{a}}C_{1\text{kHz}}k_{\text{eff}}} \end{array}$$

where *f*_r_ and *f*_a_ were the resonance and antiresonance frequencies, respectively; *C*_1kHz_ was the free capacitance of the sample at 1 kHz, which was far away from the resonance frequency; and *k*_eff_ was the effective electromechanical coupling factor, which could be obtained by:

(9) $$\begin{array}{c} k_{\text{eff}}=\sqrt{\frac{f_{\text{a}}^{2}-f_{\text{r}}^{2}}{f_{\text{a}}^{2}}} \end{array}$$

**Figure 2:**
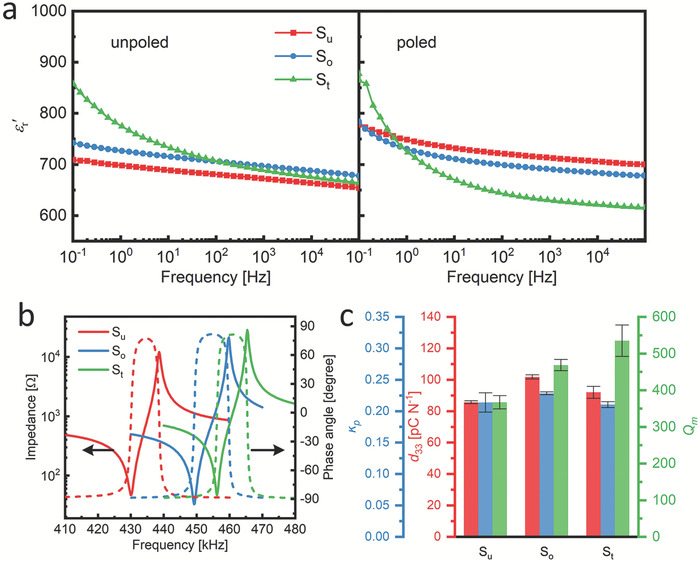
a) Real‐part relative permittivity of the S_u_, S_o_, and S_t_ samples as a function of frequency in the unpoled and poled states. b) Impedance and phase angle of the S_u_, S_o_, and S_t_ samples near the fundamental resonance frequency of the planar vibration mode. c) Electromechanical property parameters: piezoelectric coefficient, *d*_33_, planar electromechanical coupling factor, *k*_p_, and mechanical quality factor, *Q*_m_, of the S_u_, S_o_, and S_t_ samples.

The planar electromechanical coupling factor, *k*_p_, could be determined graphically from the relationship between *k*_p_/*k*_eff_ and *k*_eff_.^[^ ^36^ ^]^ All the electromechanical parameters (*d*_33_, *k*_p_, and *Q*_m_) were measured on three samples for each aging condition, and the error bars in **Figure** **2**c denote the standard deviation of the measured values for the same aging condition.

## 3 Results and Discussion

The equilibrium pseudo‐binary phase diagram of the (1−*x*)BaTiO_3_‐*x*CaTiO_3_ system (*x* represents the weight percentage of CaTiO_3_) (Figure 1a)^[^ ^37^ ^]^ indicates a strong temperature dependence in solubility of CaTiO_3_ between 1200 °C and 1500 °C. A subsolidus line divides the phase diagram into two regions: single‐phase region with a Ba‐rich solid solution (BT_ss_) and two‐phase region with the coexistence of the BT_ss_ phase and a Ca‐rich solid solution (CT_ss_). The processing of precipitation‐hardened materials can be divided into three stages: sintering, quenching, and aging (Figure 1b). The sintering process achieves densification within the single‐phase region with homogeneous distribution of elements. A quenching process follows in order to kinetically hinder the uncontrolled formation of the thermodynamically‐stable CT_ss_ phase. This results in a supersaturated solid solution, which is metastable at room temperature.^[^ ^14^, ^38^ ^]^ The temperature for the aging process^[^ ^39^ ^]^ in the two‐phase region is chosen based on both, thermodynamic as well as kinetic considerations.

The ideal microstructure for precipitation hardening is characterized by a high density of precipitates inside the grain while the size of them is in a range from several tens of nanometers to hundreds of nanometers, so that a large fraction of domain walls can be effectively hindered. According to the theory of diffusional phase transitions in solids, a reduced aging temperature leads to a larger driving force for precipitation, since the difference between the solubility and the actual solute concentration is larger. This avails the nucleation process. On the other hand, a higher temperature facilitates precipitate growth as atomic diffusion is more significant. The nucleation rate and the growth rate of precipitates can be expressed by the following equations,^[^ ^38^ ^]^

(10) $$\begin{array}{c} \frac{\text{d}N_{\mathrm{hom}}}{\text{d}t}\;\;=\;\;\omega \exp\left(-\;\;\frac{\Delta G_{\text{m}}}{kT}\right)\;\;\cdot \;\;C_{0}\exp\left(-\;\;\frac{\Delta G*}{kT}\right) \end{array}$$

(11) $$\begin{array}{c} \frac{\text{d}r}{\text{d}t}\;\;=\;\;\frac{X_{0}-X_{e}}{2\left(X_{\beta }-X_{\text{e}}\right)}\;\sqrt{\frac{D}{t}} \end{array}$$

where *N*_hom_ is the number density of the homogeneous nuclei; Δ*G*_m_ is the activation energy for atomic migration; Δ*G* ^*^ is the nucleation energy barrier, which in general decreases with decreasing temperature; *k* is the Boltzmann constant and *T* is the temperature in K; *r* is the mean radius of the precipitates; *X*_0_, *X*_e_, and *X*_b_ represent the solute concentrations in the matrix, equilibrium state, and precipitates, respectively; *D* is the interdiffusion coefficient and *t* is the duration of aging. The relationship between nucleation/growth rate and aging temperature is schematically depicted in Figure S1, Supporting Information. In addition, the aging temperature also has an influence on the nucleation on different sites. The energy barrier of heterogeneous nucleation, for example, nucleation at grain boundaries and dislocations, is reduced by these defects, and is usually lower than that of homogeneous nucleation (i.e., nucleation at defect‐free sites within grains). The homogeneous/heterogeneous nucleation ratio can be expressed by:^[^ ^38^ ^]^

(12) $$\begin{array}{c} \frac{N_{\text{het}}}{N_{\mathrm{hom}}}\;\;=\;\;\frac{C_{1}}{C_{0}}\;\;\cdot \;\;\exp\left(\frac{\Delta G_{\mathrm{hom}}^{*}-\Delta G_{\text{het}}^{*}}{kT}\right) \end{array}$$

where *N*_het_ is the number density of the heterogeneous nuclei; *C*_0_ and *C*_1_ represent the number density of the sites for homogeneous and heterogeneous nucleation, respectively; $\Delta {\text{G}}_{\text{het}}^{*}$ and $\Delta {\text{G}}_{\mathrm{hom}}^{*}$ represent the nucleation energy barrier for homogeneous and heterogeneous nucleation, respectively. A reduction in aging temperature can lead to a smaller difference between $\Delta {\text{G}}_{\text{het}}^{*}$ and $\Delta {\text{G}}_{\mathrm{hom}}^{*}$ and therefore lower heterogeneous/homogeneous nucleation ratio.

According to the abovementioned theory, the aging temperature plays an important role in the precipitate formation. An examination of this theory is provided in Figure S2, Supporting Information, where the number density, mean size, total amount, and size distribution of the precipitates in 80 wt%BaTiO_3_‐20 wt%CaTiO_3_ (BCT20) highlight the strong impact of aging temperature. The relevance of nucleation versus precipitate growth was assessed in BCT20 using three different conditions: I) as‐quenched sample without any other heat treatment (unaged sample, S_u_); II) sample aged at 1200 °C for 72 h (one‐stage aged sample, S_o_); III) sample aged at 1200 °C for 72 h and then at 1300 °C for 24 h (two‐stage aged sample, S_t_). The purpose of the two‐stage aging is to first increase the number of the precipitates within grains at the lower temperature and then to grow the precipitates at a higher temperature. The detailed temperature profile of the S_u_, S_o_, and S_t_ samples is displayed in Figure S3, Supporting Information.

Figure 1c depicts the XRD patterns of the unaged sample S_u_ and an aged sample. In order to clearly indicate the reflections of the secondary phase, an aging condition with higher aging temperature (1325 °C for 8 h, denoted by S_o_′) is selected for this comparison, which led to the highest secondary phase amount. The comparison of XRD patterns of the S_u_ and S_t_ samples are depicted in Figure S4, Supporting Information. The star symbols mark the reflections arising from the CT_ss_ phase, which is absent in the unaged sample and present in the aged sample. This confirms that the single‐phase state is achieved after quenching (within detection limits) and the CT_ss_ phase is successfully formed during the aging process. The {110}_PC_ reflections of the BT_ss_ phase shift to lower angles after aging, indicating an expansion of the matrix lattice. This can be attributed to the lower Ca content in the BT_ss_ phase due to the formation of the Ca‐rich phase (Figure S4, Supporting Information). Since Ca^2+^ has a smaller ionic radius than Ba^2+^, a larger lattice constant for the BT_ss_ with lower Ca content can be rationalized, as also evidenced in ref. ^[^ ^40^ ^]^. Figure 1d,e provides the SEM images in BSE mode of the S_u_ and the S_t_ samples, respectively. Larger areas are represented in Figure S5, Supporting Information. The CT_ss_ particles can be visualized with dark grey contrast in the SEM images of the aged sample. The size of the CT_ss_ particles varies from submicron to a few microns. Both the XRD and SEM results suggest that a homogeneous single phase has been achieved in the unaged sample and the precipitates with CT_ss_ phase emerge thereafter.

**Figure 3:**
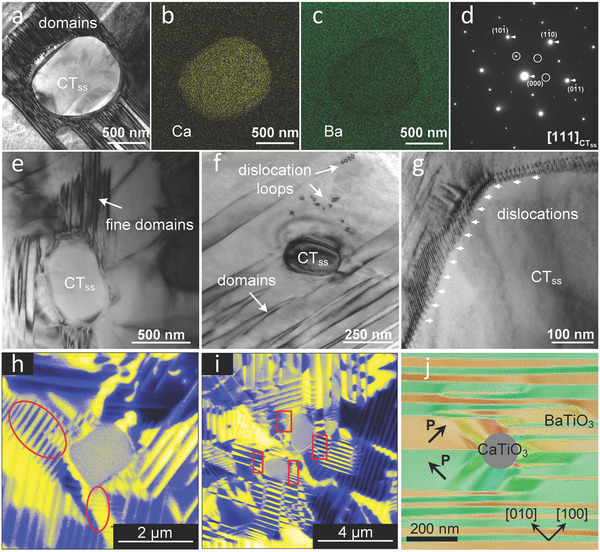
a) Bright‐field TEM image of a region in the vicinity of a precipitate and corresponding EDS mapping of b) Ca and c) Ba, respectively. d) The SAED pattern of the precipitate shown in (a). Bright‐field TEM images of e,f) the S_o_ sample and g) the S_t_ sample. h,i) PFM images of the S_t_ sample. j) Phase‐field simulation of the domain structure near a precipitate.

**Figure** **3** presents a focus on local chemistry and microstructural details around specific precipitates. A bright‐field TEM image of an intragranular precipitate and the corresponding EDS mapping of Ca and Ba are featured in Figure 3a–c. Enhancement of the Ca concentration and Ba deficiency can be observed in the precipitate, compared to the surrounding matrix, which confirms that the precipitate is of CT_ss_ phase. The electron diffraction (SAED) pattern along the [111]_PC_ zone axis of a selected area in the precipitate (Figure 3d) exhibits strong dominant reflections accompanied with weak superlattice reflections, as indicated by the arrows and circles, respectively. The strong reflections confirm that CT_ss_ has a perovskite structure. The weak superlattice reflections present at ½($10\bar{1}$), ½($1\bar{1}0$), and ½($0\bar{1}1$) indicate the existence of in‐phase tilting in the CT_ss_ precipitate.^[^ ^41^, ^42^ ^]^ Dark field TEM images of the CT_ss_ precipitate obtained using ½($10\bar{1}$), ½($1\bar{1}0$), and ½($0\bar{1}1$) superlattice reflections are displayed in Figure S6, Supporting Information.

Several structural features related to precipitates are highlighted in TEM images in Figure 3e–g: I) Change of the local domain structure: the domain pattern terminates in wedge‐shaped ends in the vicinity of the precipitate (Figure 3e,f). Regions with high domain wall density (i.e., finer domain structure) are formed near precipitates (Figure 3e); II) Emergence of dislocations: dislocation loops emerge near the precipitate (Figure 3f),^[^ ^43^ ^]^ and some dislocations are found at the precipitate/matrix interface (Figure 3g). For the fine domains near the precipitates, a similar phenomenon was observed near the grain boundaries in polycrystalline PZT and was related to increased microstrain.^[^ ^44^ ^]^ A decrease in domain size adjacent to Ag intragranular nanoparticles has also been observed in Pb(Zn_1/3_Nb_2/3_)_0.20_(Zr_0.50_Ti_0.50_)_0.80_O_3_/6 vol% Ag composites,^[^ ^45^ ^]^ while ZrO_2_ inclusions have been reported to introduce internal stresses and microcracks in PZT matrix, which inhibited domain wall movement.^[^ ^46^ ^]^ Similarly, the regions with fine domains can be attributed to the misfit strain at the precipitate/matrix interface, which arises from the difference in lattice parameters, spontaneous strain, and thermal expansion coefficients of the CT_ss_ and BT_ss_ phases.^[^ ^47^, ^48^, ^49^ ^]^ The dislocation loops appearing in the vicinity of the precipitates may be attributed to local Ca deficiency, since Ca ions have been depleted in the matrix in order to form the precipitates.

Complementary PFM images of the S_t_ sample are presented in Figure 3h,i. Precipitates were identified in PFM by the absence of a piezoelectric response. Analogous to the TEM measurements, an enhanced density of domain walls is resolved in the vicinity of a precipitate (red circles in Figure 3e) and the termination of domains at the precipitate/matrix interface (red squares in Figure 3f) can be observed in the PFM images. Please note that the domain structure also depends on the grain orientation and viewing direction, therefore a fine domain structure is not observed for all precipitates using this 2D imaging methodology.

The fine domain structure near a precipitate was further evidenced by phase‐field simulation, as highlighted in Figure 3j. Similar to the experimental observation, the precipitates alter the local structure with concurrent local refinement of the domain pattern. The simulation also indicates that the domain refinement is caused by a relaxation of the local electrostatic free energy. The spatial electrostatic free energy distributions in the refined domains near the precipitate is depicted in Figure S7b, Supporting Information, as contrasted to the case without domain refinement in Figure S7a, Supporting Information, obtained by first simulating the domain structure in a pure BaTiO_3_ and then adding a CaTiO_3_ precipitate. The electrostatic free energy density is extremely high (≈4 MJ m^−3^) around the precipitate/matrix interface without the fine domain structure due to the net bound charges at the interface, while it is substantially reduced by the formation of the fine domain structure. The average electrostatic free energy densities without and with the fine domain structure are 0.24 and 0.19 MJ m^−3^, respectively. The simulated domain configuration in a larger region around a precipitate is provided in Figure S7c, Supporting Information. We approximated the area of the fine‐domain region induced by the presence of a precipitate with a diameter of 130 nm to be 4 μm^2^, consistent with that observed in the PFM measurement.

**Figure 4:**
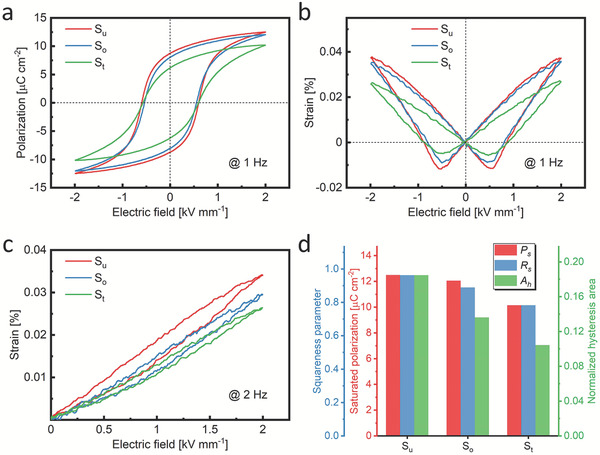
a) Bipolar polarization hysteresis loops of the S_u_, S_o_, and S_t_ samples. b) Bipolar and c) unipolar strain hysteresis loops of the S_u_, S_o_, and S_t_ samples. d) Saturated polarization *P*_s_, squareness parameter *R*_s_, and the hysteresis area of the normalized unipolar strain loops *A*_h_ of different samples.

The introduction of precipitates inevitably alters the static domain structure of the ferroelectric ceramic. This strong modification is expected to impact in a similar fashion the dynamic properties, which are highlighted next. The large‐signal properties of the S_u_, S_o_, and S_t_ samples are characterized by the polarization and strain hysteresis loops. The bipolar polarization and strain hysteresis loops of the S_u_, S_o_, and S_t_ samples (**Figure** **4**a,b) are featured next to the unipolar strain hysteresis loops in Figure 4c. It can be found that the saturated polarization, remanent polarization, and maximum strain under both bipolar and unipolar electric fields are consistently decreased from unaged (S_u_) to one‐stage aged (S_o_) to two‐stage aged (S_t_) samples. For the aged samples, two factors should be considered regarding the mechanisms of their macroscopic property change. On the one hand is the compositional change in the matrix phase (intrinsic effect). The matrix phase dominates the dielectric, ferroelectric, and piezoelectric properties of the composite rather than the precipitate phase, since the matrix usually represents a volume fraction in excess of 90% and the precipitates are not ferroelectric. On the other hand, the effect of the precipitates on the domain wall movement (extrinsic effect) should also be addressed.

In order to quantify the effect of the matrix with reduced content of Ca due to precipitation, specimens with varying Ca content in the composition range between 16 and 20 wt% CaTiO_3_ (Figure S8, Supporting Information) were prepared. Results for polarization and strain hysteresis loops for the respective single‐phase BCT solid solution demonstrate that saturated polarization and maximum strain increase with decreasing Ca content. The S_t_ sample has the largest amount of precipitates, hence the lowest Ca content in the matrix. However, it possesses the lowest remanent polarization and strain, indicating that the reduction of polarization and strain is not due to the compositional change in the matrix, but is triggered by the influence of the precipitates.

The bipolar strain hysteresis loops (Figure 4b) allow quantification of negative strain as a signature for non‐180° domain wall motion.^[^ ^50^ ^]^ The negative strain in the S_t_ sample is the smallest. As the negative strain is consistently reduced with increasing aging treatment, this measure suggests that non‐180° domain wall motions are suppressed in the aged samples.

The electromechanical loss can be quantified from unipolar loops in Figure 4c by assessing the normalized hysteresis area, *A*_h_, of the unipolar strain loops.^[^ ^51^ ^]^ This quantity is obtained by normalizing the strain loops with their maximum strain values and then calculating the closed area of the loops. The normalized hysteresis areas follow the trend of S_u_ > S_o_ > S_t_ (Figure 4d), indicating that aged samples have lower electromechanical loss under a low‐frequency, large‐signal electric field.

The empirical squareness parameter, *R*_s_, describes the shape of the loops and estimates both switching as well as back‐switching characteristics:^[^ ^52^, ^53^ ^]^

(13) $$\begin{array}{c} R_{\text{s}}\;\;=\;\;\frac{P_{\text{r}}}{P_{\text{s}}}\;\;+\;\;\frac{P_{1.1E_{\text{c}}}}{P_{\text{r}}} \end{array}$$

where *P*_r_, *P*_s_, and *P*_1.1_ *_E_*_c_ denote the remanent polarization, saturated polarization, and the polarization at 1.1 times of the coercive field, respectively. The *R*_s_ values of different samples are reduced with increasing aging treatment (Figure 4d). The back‐switching appears to be affected to a smaller degree. The more slanted curve of the aged samples is suggested to arise from a wider distribution of local switching electric fields, which is due to the broad spatial distribution of precipitates where the domain walls are pinned. The microstrain can act as a restoring force for switched domains and the high‐domain‐wall‐density regions affected by the microstrain can have higher switching fields, which was evidenced in PZT samples by a previous PFM study.^[^ ^44^ ^]^ In contrast to the properties featured in Figure 4d, aging treatment has only little influence on the coercive field, *E*_c_, as outlined in Table S1, Supporting Information.

The small‐signal properties of the S_u_, S_o_, and S_t_ samples are displayed in Figure 2. The real part of the relative permittivity ε′_r_ as a function of frequency at room temperature is revealed in Figure 2a. For the unpoled state, the aged samples exhibit a slight enhancement in relative permittivity over the whole measured frequency range. Moreover, a significant increase in the low‐frequency relative permittivity can be observed in the S_t_ sample. For the poled state, in addition to the increase at low frequency, the high‐frequency relative permittivity decreases with increasing aging degree (i.e., S_u_ > S_o_ > S_t_).

In general, the increase in the low‐frequency relative permittivity can be attributed to two mechanisms: one is an increase in conductivity of the material and the other is the Maxwell–Wagner effect (i.e., interfacial polarization), which is related to an increase in the number of interfaces between phases with different conductivity.^[^ ^8^ ^]^ For the former mechanism, the conductivity increase in ferroelectric ceramics is usually related to an introduction of charged defects, which can act as additional mobile charge carriers. In order to examine whether the enhanced low‐frequency relative permittivity is due to the long‐term aging process, a supplemental experiment was conducted: the aged sample was heated to 1500 °C and kept for 8 h followed by air quenching (requenched sample). The ε′_r_–*f* spectra of the unaged, aged, and requenched samples are contrasted in Figure S9, Supporting Information. It can be found that the ε′_r_–*f* behavior of the requenched sample is mostly reversed to the unaged state, suggesting that the long‐term aging treatment does not contribute to noticed salient effects in tuning the properties. Therefore, it is demonstrated that the rapid increase in the low‐frequency permittivity is dominantly attributed to the Maxwell–Wagner effect (space charge polarization) arising from the precipitates. The high‐frequency permittivities (kHz–MHz range) can be contributed by both the intrinsic (lattice response) and extrinsic (domain wall motion) effects. The ε′_r_–*f* spectra of unaged and unpoled BCT samples with different Ca content are depicted in Figure S10, Supporting Information, from which it can be seen that the compositional change has negligible influence on the ε′_r_–*f* behavior. Thus, it is reasonable that the high‐frequency permittivities of the S_u_, S_o_, and S_t_ samples are comparable and the differences are less than 7%. In addition, a difference in the dielectric response of the unpoled and poled states can be noticed from the permittivity frequency spectra in the high‐frequency range. The aging treatment has led to a decrease in the high‐frequency permittivity at poled state, while this influence is negligible at unpoled state.

Frequency‐dependent impedance and phase angle spectra of the poled samples are obtained near the resonance frequency to characterize their electromechanical properties.^[^ ^36^ ^]^ All samples had a disk shape; therefore, the radial vibration mode was adopted according to the European standard.^[^ ^54^ ^]^ The impedance spectra (Figure 2b) reveal a resonance frequency of the samples at around 450 kHz. The piezoelectric coefficient, *d*_33_, planar electromechanical coupling factor, *k*_p_, and mechanical quality factor, *Q*_m_, of the S_u_, S_o_, and S_t_ samples are displayed in Figure 2c. The poling degree of the samples may have an influence on their electromechanical properties. Thus, all the samples were poled at an electric field of 4 kV mm^−1^ (approximately seven times the *E*_c_) before the measurement to ensure that they are fully poled. Also, the phase angles of these samples between the resonance and antiresonance frequencies reach a level of ≈81°, which is close to the ideal 90° and is an indicator that the poling degrees of all samples are relatively high. The *d*_33_ and *k*_p_ have similar trends: after one‐time aging, the S_o_ sample has enhanced *d*_33_ and *k*_p_, while two‐stage aging (S_t_ sample) reduces these values, then again comparable to the unaged, S_u_ sample. For the mechanical quality factor, *Q*_m_, all the aged samples have enhanced values, with the S_t_ sample the highest *Q*_m_ value increased by 50% compared to that of the S_u_ sample.

Similarly, the *d*_33_, *k*_p_, and *Q*_m_ are contrasted in single‐phase BCT with 0.16 < CaTiO_3_ wt% < 0.20 (Figure S11, Supporting Information). It is found that with decreasing Ca content, the *d*_33_ and *k*_p_ increase while the *Q*_m_ decreases. This indicates that the increase of *Q*_m_ in the aged samples is attributed to the effect of precipitates, rather than to the decrease in Ca content in the matrix. The increased *Q*_m_ in the aged samples can be understood as a suppression of domain wall motion, as suggested from the large‐signal polarization and strain hysteresis loops and is correlated to the microstructural modifications around the precipitates. In addition, a measurement of permittivity at subcoercive fields as a function of field amplitude (Rayleigh measurement, Figure S12, Supporting Information) also supports that the irreversible contribution to permittivity (i.e., irreversible domain wall motion) is smaller in the S_o_ and S_t_ samples.^[^ ^55^ ^]^

The changes of *k*_p_ and *d*_33_ describe competing tendencies, as an increase in *Q*_m_ is usually accompanied with a decrease in *k*_p_ and *d*_33_.^[^ ^56^, ^57^ ^]^ It is suggested that the *k*_p_ and *d*_33_ enhancement in the aged samples arises from the intrinsic contribution, as the enhanced *Q*_m_ suggests a suppression of the extrinsic contribution (i.e., domain wall motion) in the aged sample. Therefore, the continuous aging treatment prompts enhanced mechanical quality factor through enhanced domain wall pinning by the precipitates. This should also reduce the piezoelectric coefficient, *d*_33_, and the planar coupling factor, *k*_p_. However, this decrease in the extrinsic properties is more than compensated by the enhancement in intrinsic piezoelectric effect in the aged samples (Figure S11, Supporting Information). Therefore, the one‐stage aged samples, S_o_, provide the highest values for these thermal treatment conditions. Furthermore, the space charges accumulated at the precipitate/matrix interfaces in the poled samples may also have an influence on the piezoelectric response. Analogous to the charge compensation model proposed in the NBT‐6BT:ZnO composites,^[^ ^20^ ^]^ the space charges at the interfaces could depress the depolarization field in the matrix grains and stabilize the poled domain structure. Therefore, the local spontaneous polarization increases, which contributes to larger *d*_33_ and *k*_p_, and the stabilized domain structure may contribute to the *Q*_m_ enhancement.

Precipitation hardening is a generic approach and can be implemented in other ferroelectric ceramics. Suitable material systems can be classified into two categories: I) solid solutions with temperature‐dependent solubility, and II) doped ferroelectrics with the dopant concentration beyond the solubility limit, which usually can be enhanced by increasing the sintering temperature.

## 4 Conclusions

Precipitate formation in the model system BaTiO_3_ alloyed with CaTiO_3_ has been demonstrated to:

- Yield domain refinement in the vicinity of the precipitate.
- Increase the mechanical quality factor by 50% and reduce the hysteretic losses
- Unlike in acceptor‐hardened piezoelectrics, piezoelectric coefficient and electromechanical coupling factor are retained after precipitate hardening.

The required thermal treatment to induce precipitation can easily be implemented into the industrial production process. Refinements of the microstructure toward a finer and better‐dispersed distribution of precipitates are expected with wide adoption of this simple process in all other relevant ferroelectric materials and should lead to strong property enhancements for lead‐free as well as lead‐containing hard ferroelectrics.

## Conflict of Interest

The authors declare no conflict of interest.

## Supporting information

Supporting Information

## Data Availability

Research data are not shared.
