# Supplementary material for: Precipitation Hardening in Ferroelectric Ceramics
Source: Adv Mater. 2021 Jul 24;33(36):2102421. doi: 10.1002/adma.202102421 (PMC11469274; doi:10.1002/adma.202102421)
Supplement: Supplementary file 1 — Supporting Information [file ADMA-33-2102421-s001.pdf]

© 2021 Wiley-VCH GmbH

ADVANCED MATERIALS

Supporting Information

for *Adv. Mater.,* DOI: 10.1002/adma.202102421

Precipitation Hardening in Ferroelectric Ceramics

Changhao Zhao, Shuang Gao, Tiannan Yang, Michael Scherer, Jan Schultheiß, Dennis Meier, Xiaoli Tan, Hans-Joachim Kleebe, Long-Qing Chen, Jurij Koruza, and Jürgen Rödel**

Supplemental Information

Precipitation hardening in ferroelectric ceramics

Changhao Zhao, Shuang Gao, Tiannan Yang, Michael Scherer, Jan Schultheiß, Dennis Meier, Xiaoli Tan, Hans-Joachim Kleebe, Long-Qing Chen, Jurij Koruza, and Jürgen Rödel**

Dr. C. Zhao, Dr. S. Gao, M. Scherer, Prof. H.-J. Kleebe, Dr. J. Koruza, Prof. J. Rödel
Department of Materials and Earth Sciences, Nonmetallic Inorganic Materials
Technical University of Darmstadt
Alarich-Weiss-Straße 2, 64287 Darmstadt, Germany
Email: koruza@ceramics.tu-darmstadt.de
Email: roedel@ceramics.tu-darmstadt.de

Dr. J. Schultheiß, Prof. D. Meier
Department of Materials Science and Engineering
Norwegian University of Science and Technology
7034 Trondheim, Norway

Dr. T. Yang, Prof. L.-Q. Chen
Materials Research Institute and Department of Materials Science and Engineering
The Pennsylvania State University
University Park, Pennsylvania 16802, USA

Prof. X. Tan
Department of Materials Science and Engineering
Iowa State University
Ames, Iowa 50011, USA

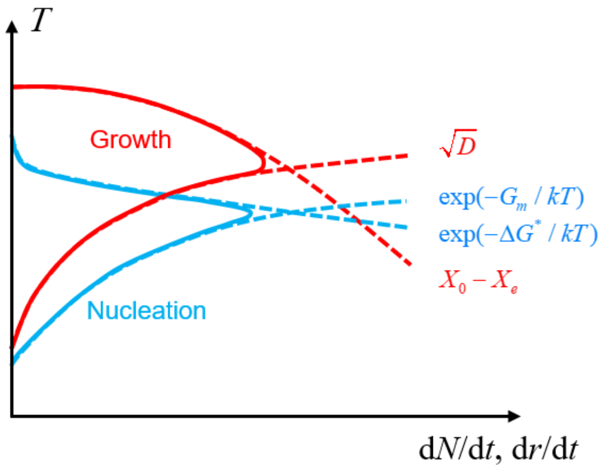

Figure S1. Temperature dependence of the precipitate nucleation rate (blue; dN/dt) and the growth rate (red; dr/dt). D represents the atomic interdiffusion coefficient; X_0 and X_e represent the solute concentration in the matrix and at equilibrium state, respectively; G_m represents activation energy for atomic migration; ΔG^* is the nucleation energy barrier.

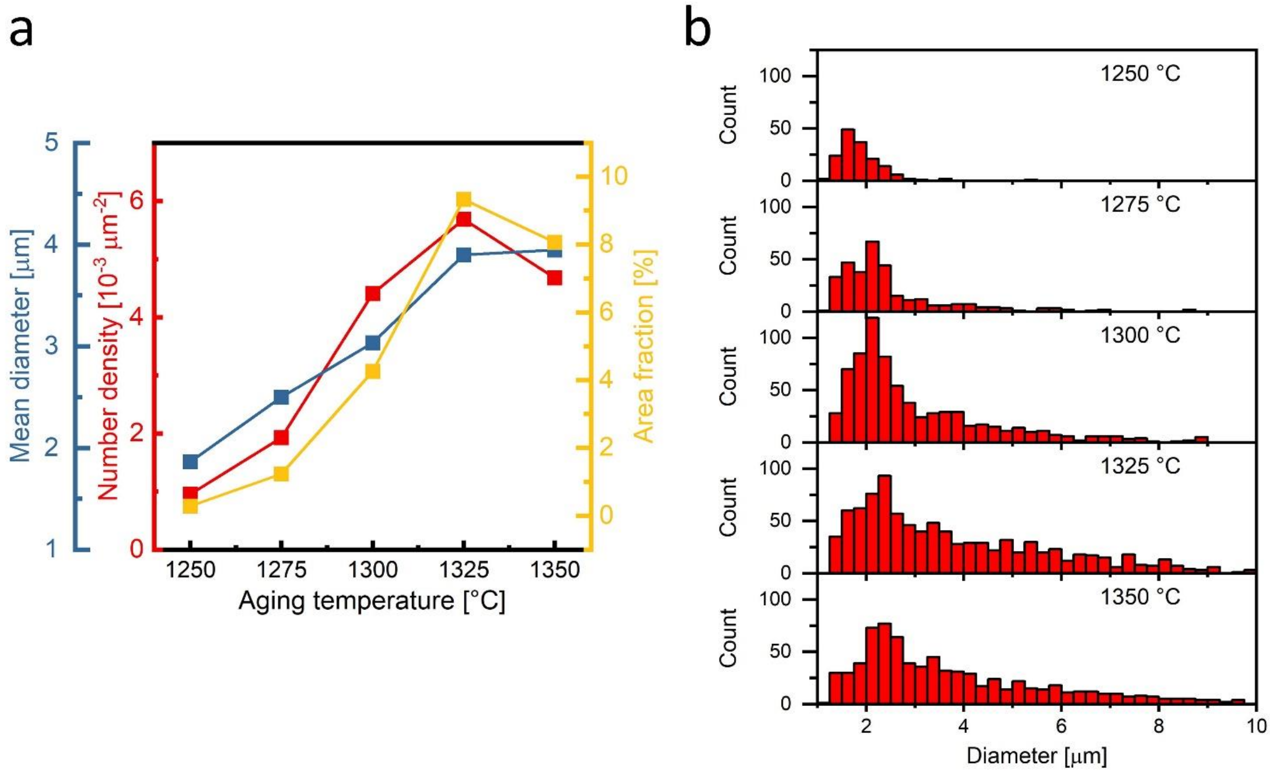

Figure S2. Quantification of precipitates in BCT20 samples treated at different aging temperatures between 1250 °C and 1350°C (one-step aging). (a) Area number density, mean diameter, and area fraction. (b) Size distribution of precipitates in the BCT20 aged at different temperatures for 8 h. The statistical data was obtained from SEM images using the back-scattered electron imaging mode. A total area of about 0.166 mm^2 was analyzed.

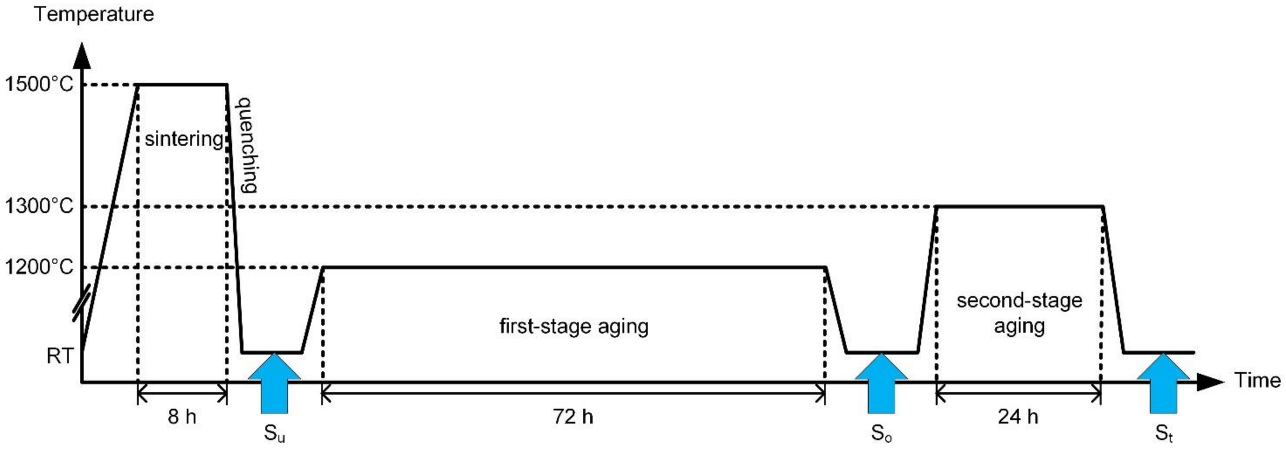

Figure S3. Temperature profile of the processing of the BCT20 samples S_u, S_o, and S_t. The samples were obtained after the quenching process, first-stage aging, and second-stage aging, respectively.

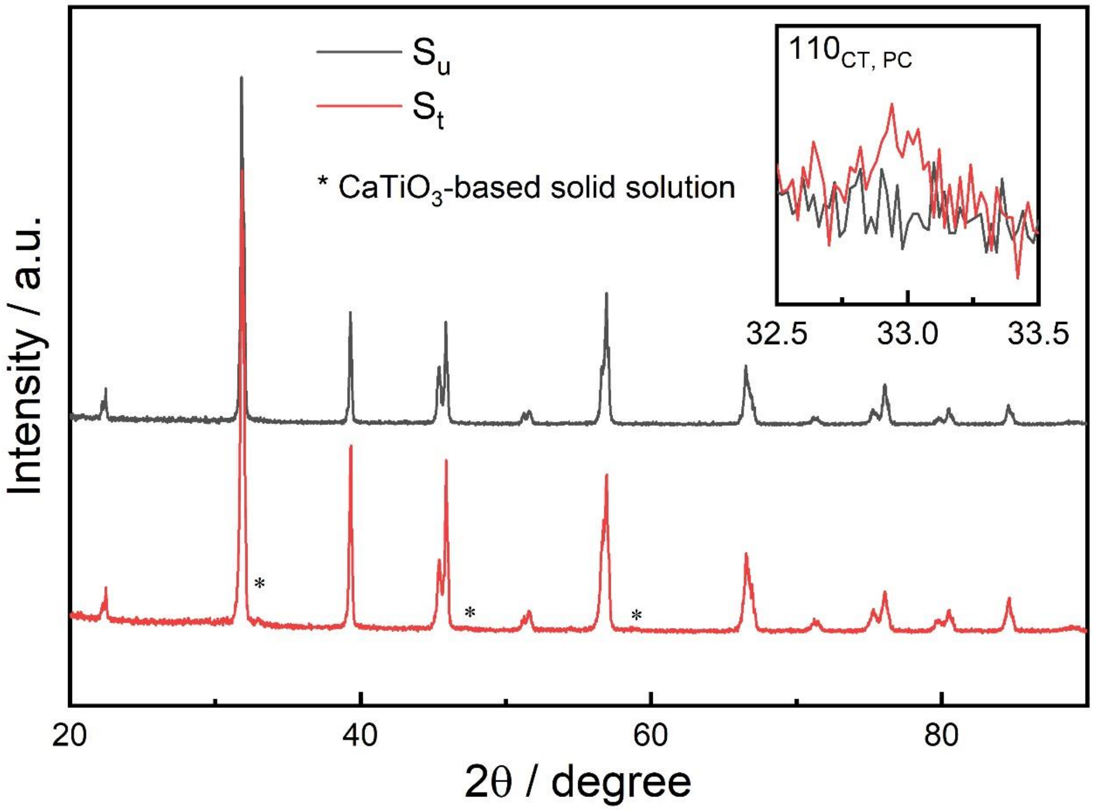

Figure S4. XRD patterns of the S_u and S_t samples. The star symbols represent the reflections from precipitates of the CT_{ss} phase in the S_t sample.

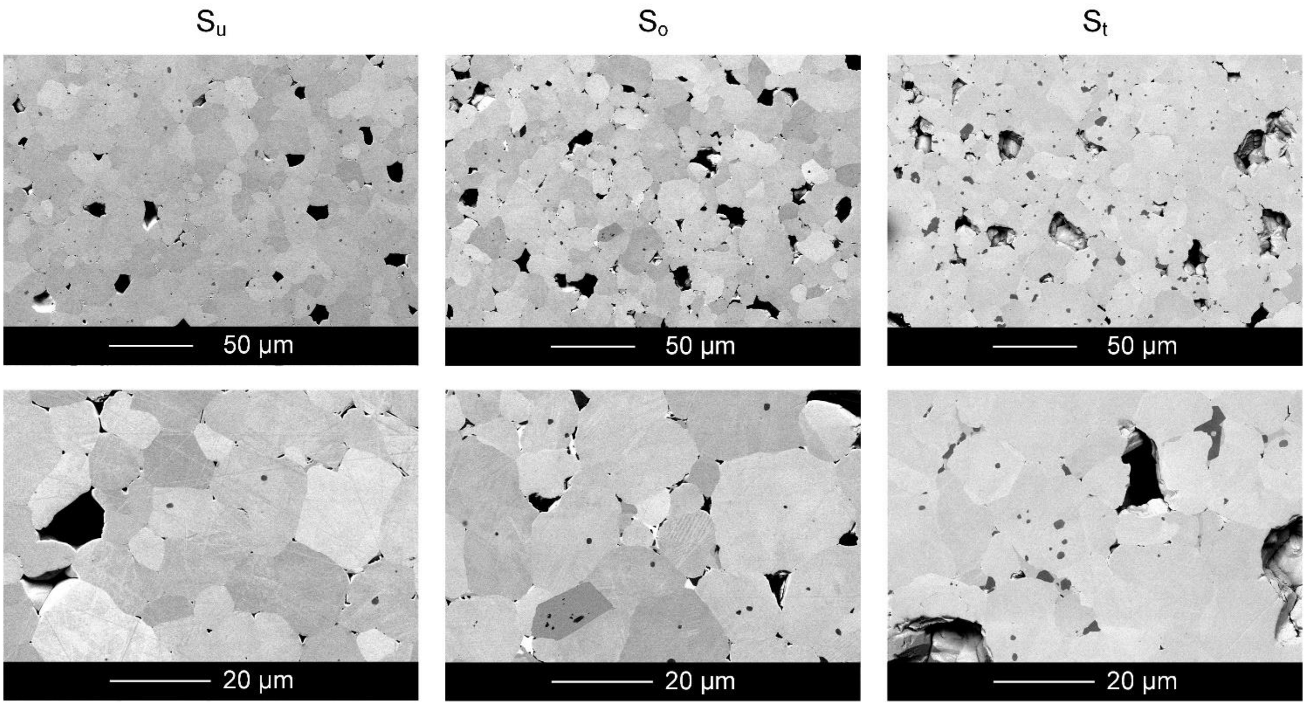

Figure S5. SEM images (back-scattered electron mode) of the polished S_u, S_o and S_t samples with different magnifications. While a small amount of precipitates can still be observed in the S_u sample due to the limited cooling rate in air quenching, the number of intragranular precipitates increases strongly in the S_o sample. Compared to the S_o sample, the S_t sample has precipitates with larger size, and the precipitates at the grain boundaries become significant.

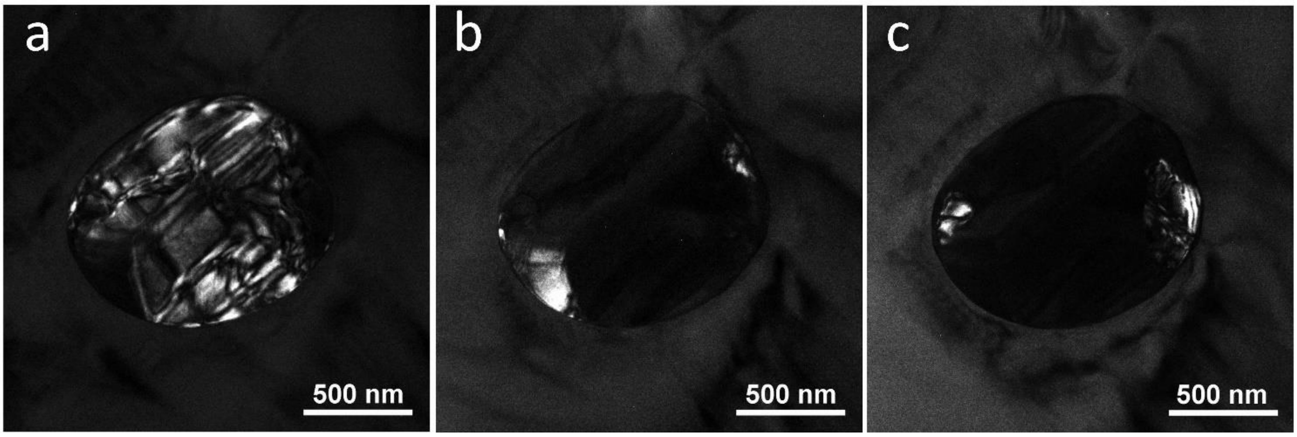

Figure S6. Dark field TEM images obtained using (a) ½ $(10\bar{1})$, (b) ½ $(1\bar{1}0)$, and (c) ½ $(0\bar{1}1)$ superlattice reflections, which are circled in Figure 2d. The bright area in Figure S6a is much larger than that in Figure S6b and S6c, which is consistent with the SAED pattern in Figure 2d that, the intensity of ½$(10\bar{1})$ reflection is clearly stronger than the intensity of ½$(1\bar{1}0)$ and ½$(0\bar{1}1)$.

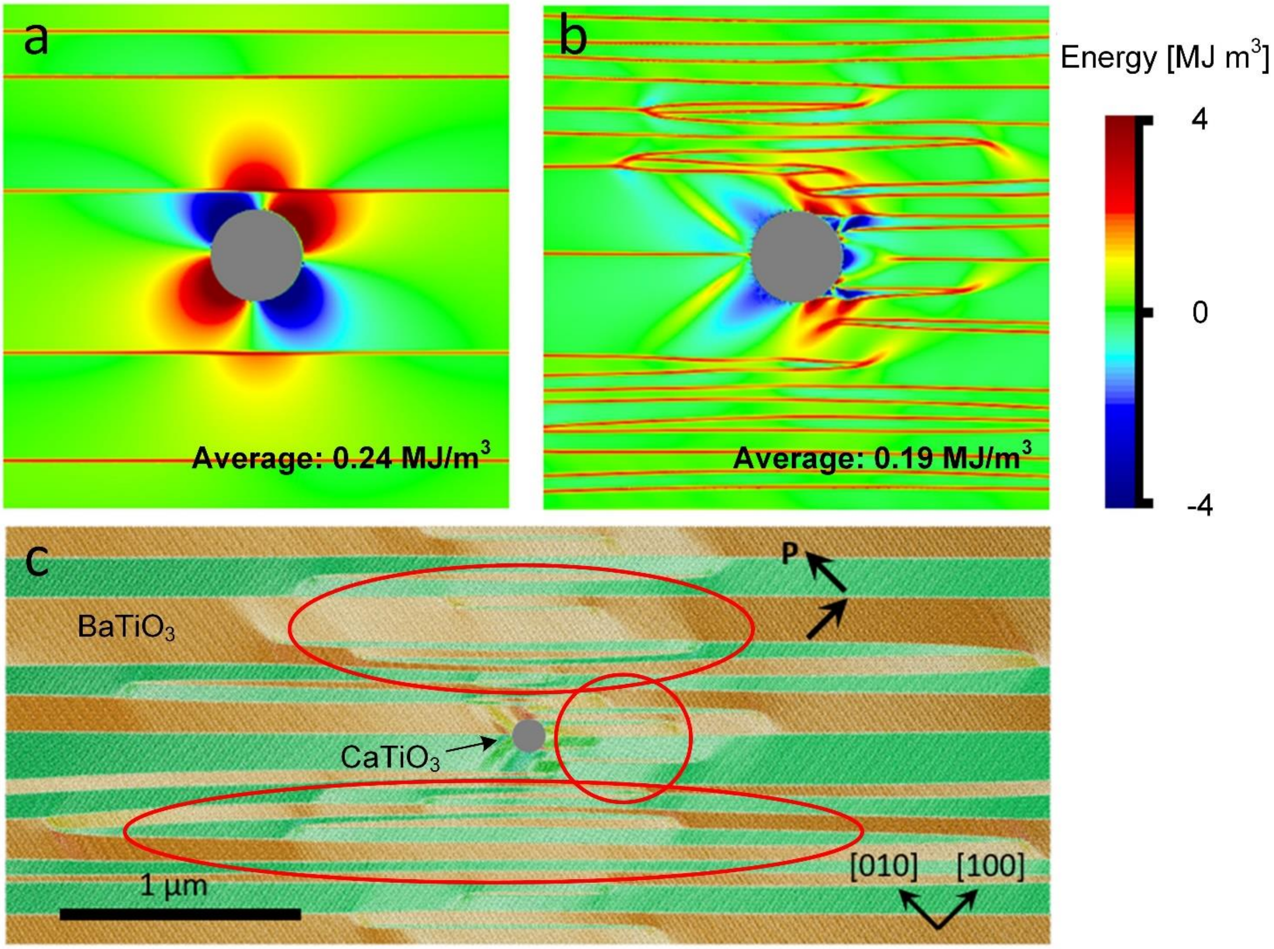

Figure S7. Calculated distributions of the electrostatic free energy density in the (a) absence and (b) presence of the fine domain structure. The electrostatic free energy density is given by $f_{elec} = -\varepsilon_0\varepsilon^b E^2/2 - \mathbf{E}\cdot\mathbf{P}$, where ε^b is the background dielectric constant and $\mathbf{E}$ is the local electric field. (c) Large-area phase-field simulation of the domain configuration near a precipitate.

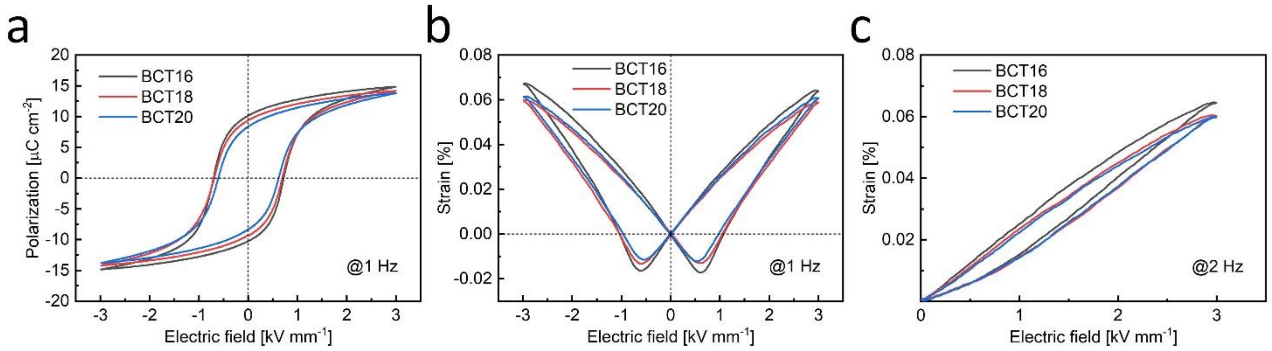

Figure S8. Bipolar (a) polarization and (b) strain hysteresis loops, and (c) unipolar strain loops of unaged BCT samples with different compositions ($CaTiO_3$ wt.% = 0.16, 0.18, and 0.20)

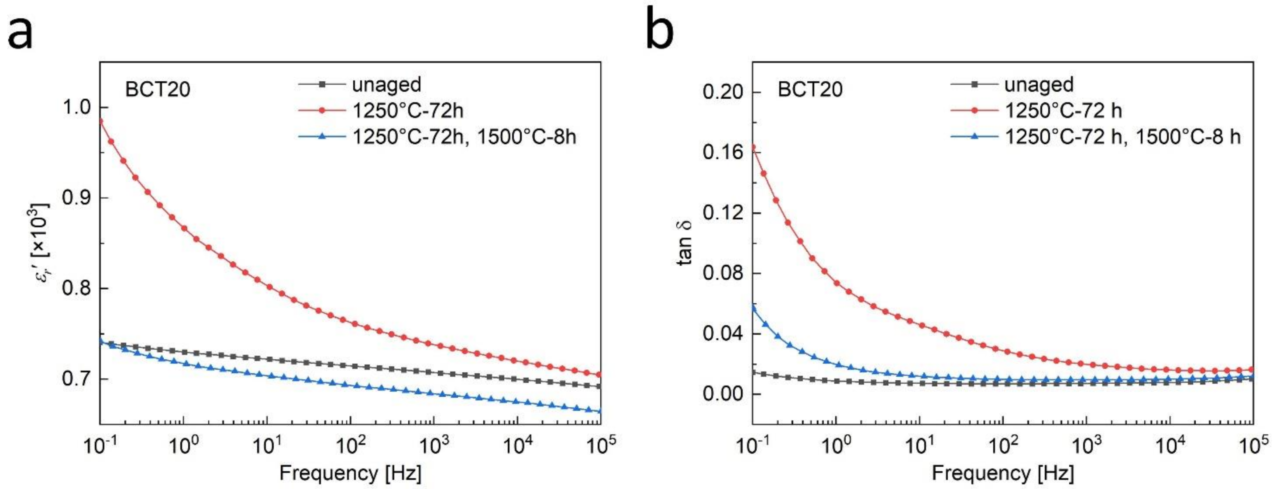

Figure S9. Real part of the permittivity and dielectric loss as a function of frequency of the BCT20 samples which are unaged, aged at 1200°C for 72 h, and aged at 1200°C for 72 h followed by a quenching from 1500°C.

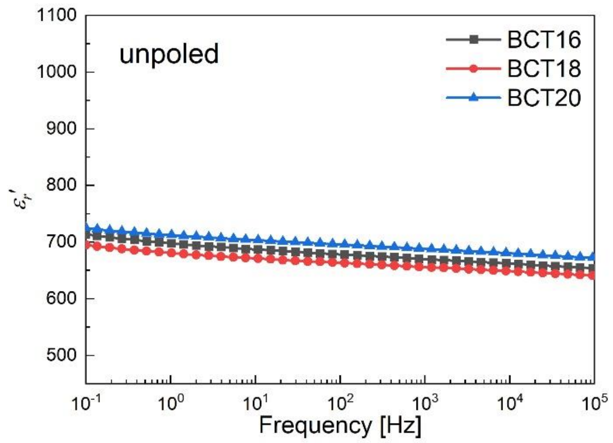

Figure S10. Permittivity of unpoled BCT samples with different Ca content as a function of frequency. The unaged BCT samples without precipitates have comparable permittivity-frequency behaviors in the Ca content range of 16 wt.% to 20 wt.%, indicating that the compositional changes in BCT (Ba/Ca ratio) have negligible influence on permittivity.

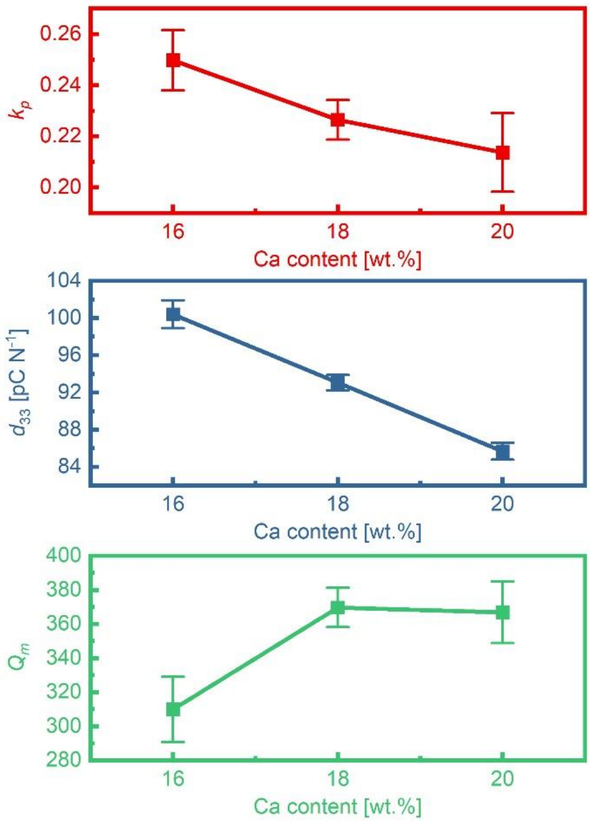

Figure S11. Planar electromechanical coupling factor k_p, longitudinal piezoelectric coefficient d_{33}, and small-signal mechanical quality factor in planar vibration mode Q_m of BCT samples with different compositions.

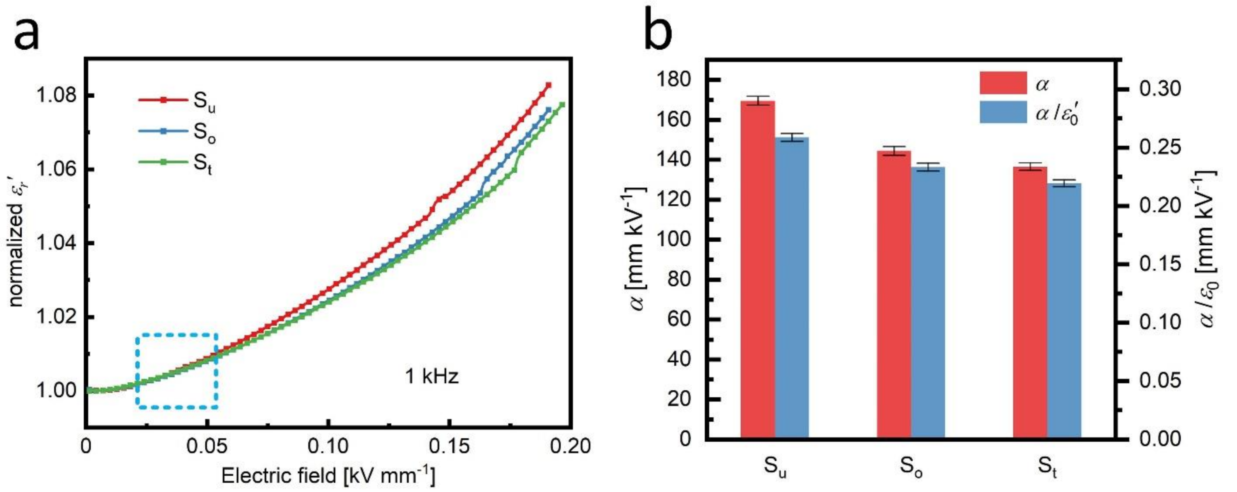

Figure S12. Rayleigh measurement results of the S_u, S_o and S_t samples. According to the Rayleigh theory, the permittivity at subcoercive field can be expressed as: $\varepsilon_r'(E)=\varepsilon_0'+\alpha E$, where $\varepsilon_r'(E)$ is the permittivity at a field with amplitude of E; ε_0' is the permittivity of the sample at extremely small field, where the contributing mechanisms are in principle intrinsic lattice effect and reversible domain wall motion; α is the nonlinear dielectric coefficient, which describes the field-amplitude dependence of the permittivity and results from irreversible domain wall motion. (a) Normalized permittivity as a function of the field amplitude. The normalization factor is the permittivity at the smallest field, which is close to ε_0'; (b) The α values (red bars) extracted from linear fitting of the ε_r'-E curve in the region indicated by the blue dashed square, as well as the ratio of α to ε_0' (blue bars).

Table S1. Coercive fields E_c of the S_u, S_o, and S_t samples. The E_c values were extracted from the polarization hysteresis measurement with a bipolar electric field with $|E_{max}|$ = 2 kV/mm.

Samples	S_u	S_o	S_t
E_c [kV mm^{-1}]	0.60	0.54	0.58
